# Silencing of circular RNA ANRIL attenuates oxygen–glucose deprivation and reoxygenation-induced injury in human brain microvascular endothelial cells by sponging miR-622

**DOI:** 10.1186/s40659-020-00295-2

**Published:** 2020-07-02

**Authors:** Su Jiang, Gaonian Zhao, Jun Lu, Min Jiang, Zhenggang Wu, Yujing Huang, Jing Huang, Jinghua Shi, Jing Jin, Xinxuan Xu, Xuehua Pu

**Affiliations:** 1 Department of Rehabilitation, Taizhou People’s Hospital, Taizhou, Jiangsu 225300 People’s Republic of China; 2 Department of Neurosurgery, Taizhou People’s Hospital, Taizhou, Jiangsu 225300 People’s Republic of China; 3 Department of Neurology, Taizhou People’s Hospital, Taizhou, Jiangsu 225300 People’s Republic of China; 4 Department of Critical Care Medicine, Taizhou People’s Hospital, Taizhou, Jiangsu 225300 People’s Republic of China

**Keywords:** Oxygen–glucose deprivation and reoxygenation, Human brain microvascular endothelial cells, circ_ANRIL, miR-622

## Abstract

**Background:**

Circular RNA (circRNA) is highly expressed in the brain tissue, but its molecular mechanism in cerebral ischemia–reperfusion remains unclear. Here, we explored the role and underlying mechanisms of circRNA antisense non-coding RNA in the *INK4* locus (circ_ANRIL) in oxygen–glucose deprivation and reoxygenation (OGD/R)-induced cell injury.

**Results:**

The expression of circ_ANRIL in OGD/R-induced human brain microvascular endothelial cells (HBMECs) was significantly up-regulated, while that of miR-622 was significantly down-regulated. Overexpression of circ_ANRIL significantly inhibited the proliferation of OGD/R-induced HBMECs and aggravated OGD/R-induced cell apoptosis. Moreover, circ_ANRIL overexpression further increased the secretion of interleukin (IL)-1β, IL-6, tumor necrosis factor-α, and monocyte chemoattractant protein-1 in OGD/R-treated HBMECs. The results of bioinformatics analysis and luciferase reporter assay indicated that circ_ANRIL served as an miR-622 sponge to negatively regulate the expression of miR-622 in OGD/R-treated HBMECs. Additionally, circ_ANRIL silencing exerted anti-apoptotic and anti-inflammatory effects by positively regulating the expression of miR-622. Furthermore, inhibition of OGD/R-induced activation of the nuclear factor (NF)-κB pathway by circ_ANRIL silencing was significantly reversed by treatment with miR-622 inhibitor.

**Conclusions:**

Knockdown of circ_ANRIL improved OGD/R-induced cell damage, apoptosis, and inflammatory responses by inhibiting the NF-κB pathway through sponging miR-622.

## Background

Insufficient blood supply followed by blood reperfusion leads to ischemia and reperfusion injury, which is the cause of various complications and deaths associated with stroke, myocardial infarction, and traumatic brain injury [1]. Cerebral vascular endothelial cell injury induced by cerebral ischemia–reperfusion (CI/R) is an important cause of brain tissue damage after focal cerebral ischemia [2], which is also the initial stage of blood–brain barrier destruction [3]. The mechanism of CI/R injury, a complex pathological process, remains unclear. Current studies suggest that the pathophysiological mechanisms responsible for CI/R injury involve oxidative stress, excitatory amino acid toxicity, inflammatory response, and apoptosis. However, effective clinical drugs for the treatment of CI/R injury are still lacking. Therefore, an in depth understanding of the underlying mechanisms of CI/R injury is important for developing effective treatments.

Circular RNA (circRNA) is a class of non-coding RNAs produced by a special selective cleavage mechanism. circRNAs do not have 5′ caps and 3′ polydenylated tails, but a closed loop structure that usually consists of more than one exon [4]. The brain tissue is rich in circRNAs [5]. These circRNAs are not only related to the development, differentiation, and biological functions of the nervous system, but also play an important role in nervous system dysfunction and pathology after brain injury [6]. Recent studies have reported that the expression profile of circRNAs in brain tissues under cerebral ischemia is altered, and certain circRNAs may be involved in the pathogenesis of cerebral ischemia [7]. Hence, investigating the role of circRNAs in the pathogenesis of CI/R injury is of great significance for the early diagnosis and treatment of ischemic brain injury.

Antisense non-coding RNA in the *INK4A* locus (ANRIL) is a long-chain non-coding RNA consisting of 19 exons located at 9p21. Zhang et al. [1] showed that ANRIL is not only associated with susceptibility to atherosclerosis and hemorrhagic stroke in stroke populations, but also independently predicts the risk of stroke recurrence and cardiovascular death. Moreover, Congrains et al. [8] demonstrated that ANRIL plays an important regulatory role in cardiovascular disease and Alzheimer’s disease. Interestingly, it has been reported that ANRIL is capable of forming RNA circles, and a circular isoform of ANRIL (circ_ANRIL) is associated with the development of atherosclerosis [9]. Holdt et al. [10] showed that circ_ANRIL regulates the development of atherosclerosis by inducing p53 activation, which leads to induction of apoptosis and inhibition of proliferation. Furthermore, Song et al. [11] showed that inhibition of circ_ANRIL can prevent the development of coronary atherosclerosis by reducing vascular endothelial cell apoptosis and inflammatory response. However, the molecular mechanism of circ_ANRIL in CI/R remains to be further explored.

Recent studies have suggested that circRNAs can act as molecular “sponges” for microRNAs (miRNAs) [12]. For example, circ_CDR1as has a conserved sequence that matches miR-7, and was shown to improve insulin secretion by inhibiting miR-7 function in islet cells [13]. Additionally, circ_CDR1as exerts anti-oncogenic functions by sponging miR-135a in bladder cancer [14]. miRNAs are a class of single-stranded non-coding small RNAs of about 21–27 nucleotides in length, which are capable of inhibiting mRNA translation by binding to their 3ʹ-untranslated region (UTR). Increasing evidence suggests that abnormal expression of miRNAs is closely related to stroke [15]. miR-622 has been shown to act as a tumor suppressor in several common cancers [11, 16, 17]. Zhang et al. [17] showed that miR-622 expression was significantly down-regulated in glioma tissues and cell lines, and targeting miR-622 may be a novel therapeutic approach to block glioma invasion. Additionally, Zhou [18] demonstrated that miR-622 expression in the peripheral blood of patients with ischemic stroke was significantly down-regulated, indicating that miR-622 may act as a biomarker for predicting the risk of ischemic stroke. However, the direct involvement of miR-622 in CI/R injury has not yet been studied.

In the present study, through bioinformatics analysis (circBase [19] and StarBase v2.0 [20]), we found that circ_ANRIL has miR-622 seed matches. Based on these results, we hypothesized that circ_ANRIL may be involved in CI/R injury by inhibiting the expression of miR-622. Here, we established an in vitro CI/R injury model by exposing human brain microvascular endothelial cells (HBMECs) to oxygen–glucose deprivation and reoxygenation (OGD/R) HBMECsto investigate the role of circ_ANRIL in CI/R injury.

## Methods

### Cell culture

Primary HBMECs were purchased from Cell Systems Corp. (Kirkland, WA, USA). Cells were maintained in RPMI-1640 medium (Gibco; Thermo Fisher Scientific, Inc., Waltham, MA, USA) containing 10% fetal bovine serum (FBS; Gibco) and cultured in an incubator at 37 °C, 5% CO_2_, and 90% humidity.

### Establishment of OGD/R model

The OGD/R model was constructed as described previously [21]. In brief, complete medium with glucose was replaced with glucose-free Hank’s balanced salt solution (Invitrogen, Thermo Fisher Scientific, Inc., Waltham, MA, USA). The cells were cultured under hypoxic conditions (containing 5% CO_2_, 0.3% O_2_, and 94.7% N_2_) for 2, 4, and 8 h. Subsequently, the glucose-free medium was replaced with complete medium containing glucose and 10% FBS, and cells were placed in an incubator containing 95% air and 5% CO_2_ and incubated at 37 °C for 24 h. Cells grown under normal culture conditions were used as a control.

### CCK-8 assay

The cells were seeded in 96-well plates at a density of 2 × 10^3^ cells/well, and then cultured in a CO_2_ incubator at 37 °C for 24 h. Four repetitions were set for each well. After transfection and/or OGD/R treatment, 20 μl of CCK-8 reagent (Dojindo Molecular Technologies, Dojindo, Japan) was added to each well and incubated for 4 h at 37 °C. Be careful not to introduce bubbles to the wells, since they interfere with the optical density (OD) reading. The OD value was measured using a NanoDrop spectrophotometer (NanoDrop Technologies, Inc., Montchanin, Delaware, New Castle) at 450 nm. Before reading the plate, it is important to mix gently on an orbital shaker for 1 min to ensure homogeneous distribution of color. In this study, the restuls in the control group was calculated as 100%, and other groups were expressed as % of the control group.

### Cytotoxicity assay

The culture supernatant before and after cell lysis was collected, and the amount of lactate dehydrogenase (LDH) released into the medium was measured to determine the cytotoxicity. LDH activity was measured according to the LDH cytotoxicity assay kit (Invitrogen). All operations were carried out in strict accordance with the manufacturer’s instructions.

### Cell transfection

Cells were seeded into 6-well plates at a density of 6 × 10^4^ cells/2 ml/well and maintained in an incubator at 37 °C, 5% CO_2_. When the cells reached about 80% confluency, cell transfection was performed. The circ_ANRIL (hsa_circ_0008574) overexpression vector was synthesized by Sangon Biotech (Shanghai, China), and an empty vector (circ-NC) was used as a control. All miRNAs mimics (miR-622 mimic: 5′-ACAGUCUGCUGAGGUUGGAGC-3′, NC-mimic: 5′-UUCUCCGAACGUGUCACGUTT-3′), and miRNAs inhibitor (miR-622 inhibitor: 5′-GCUCCAACCUCAGCAGACUGU-3′, NC-inhibitor: 5′-CAGUACUUUUGUGUAGUACAA-3′) were purchased from RiboBio (Guangzhou, Guangdong, China). circ_ANRIL was knocked down using specific short interfering RNAs (siRNAs: si-circ_ANRIL: 5′-AGAATTTTGACAGTGTCCCTT-3′, si-NC: 5′TTCTCCGAACGTGTCACGT-3′) targeting the backsplice region. HBMECs were transfected with plasmids or oligonucleotides HBMECs using Lipofectamine™ 2000 (Life Technologies, Carlsbad, CA, USA), according to the manufacturer’s instructions. After 24 h of transfection, the efficiency of transfection was determined by qRT-PCR.

### qRT-PCR

Total RNA was extracted from cells using TRIzol reagent (Takara Bio, Shiga, Japan). RNA concentration was measured using the NanoDrop spectrophotometer (NanoDrop Technologies, Wilmington, DE, USA). RNA was then reverse transcribed into cDNA using the GoScript Reverse Transcription System (Promega, Madison, WI, USA). The SYBR RT-PCR kit (Takara, China) was used for mRNA quantification with specific primers. For miR-622, cDNA was generated with the miScript II RT kit (Qiagen, Hilden, Germany) using RT-U6- and miRNA-specific stem loop primers. The miScript SYBR Green PCR Kit (Qiagen) was used for miRNA quantification. All qRT-PCR reactions were performed on an ABI 7500 PCR Instrument (ABI, Bedford, MA, USA). The qRT-PCR amplification was performed under the following reaction conditions: 95 °C for 5 min; 40 cycles of 95 °C for 20 s, 58 °C for 30 s, and 72 °C for 30 s. The primers were as follows; circ_ANIRL: forward, 5′-TGTACTTAACCACTGGACTACCTGCC-3′ and reverse, 5′-TCCACCACACCTAACAGTGATGCTTG-3′; miR-622: forward, 5′-ATCCCAGGGAGACAGAGATCGAGG-3′ and reverse, 5′-AAGCTTGGTGGTGGACTTTTGGTTGT-3′; U6: forward, 5′-GGTGAAGCAGGCGTCGGAGG-3′ and reverse, 5′-GAGGGCAATGCCAGCCCCAG-3′; GAPHD: forward, 5′-GCACCGTCAAGGCTGAGAAC-3′ and reverse, 5′-TGGTGAAGACGCCAGTGGA-3′.

### Flow cytometry

Cell apoptosis was detected using an Annexin V-FITC/propidium iodide (PI) apoptosis detection kit (Invitrogen). The experimental protocol was carried out in strict accordance with the manufacturer’s instructions. Briefly, the cells were cultured in 6-well plates and cultured overnight. After being subjected to the transfection and/or OGD/R treatment as described above, cells were harvested and washed twice with phosphate buffered saline in 1.5 ml tube and 300 μl binding buffer was added to the tube. Then 5 μl Annexin V—FITC was added to the tube and incubated at room temperature in the dark for 30 min. Then, 10 μl PI solution was added to the tube and incubated for 10 min at room temperature in the dark. Cell apoptosis were analyzed using the BD FACSVerse flow cytometer (FACScan; BD Biosciences, Franklin Lakes, NJ, USA).

### Western blotting

Cells were lysed using RIPA buffer and total protein was extracted from the lysate. Total protein concentration was determined using a bicinchoninic acid assay kit (KeyGen, Nanjing, China). Protein samples were separated by electrophoresis on a 13% sodium dodecyl sulfate–polyacrylamide gel and transferred to a polyvinylidene fluoride membrane (Millipore, Billerica, MA, USA). After blocking with 5% skim milk at 25 °C for 1 h, the membrane was incubated with the following primary antibodies: anti-Bcl-2 (1:1000; Abcam, USA), anti-Bax (1:1000; Abcam), anti-P-Iκnt (1:1000; Beyotime, China), anti-Iκnt (1:1000; Beyotime, China), anti-P-p65 (1:1000; Abcam), anti-p65 (1:1000; Abcam), and anti-β-actin (1:500; Beyotime, Hangzhou, China) overnight at 4 °C. After washing with Tris-buffered saline containing 0.1% Tween 20 (TBST), the membrane was incubated with horseradish peroxidase-labeled secondary antibody IgG (1:1000; Abcam, USA) for 1 h at 37 °C. Blots were visualized with an enhanced chemiluminescence kit (Amersham, Little Chalfont, UK) and quantified using the Image Pro-Plus 6.0 analysis system (Media Cybernetics, Rockville, MD, USA). Band density values were normalized to β-actin.

### Enzyme-linked immunosorbent assay (ELISA)

Cells were treated with 100 μl precooled Radio-Immunoprecipitation assay (RIPA) lysis buffer (2 ml/g) for lysis of 15 min at 4 °C, and after centrifugation at 2500 r/min for 15 min at 4 °C, the supernatant was collected. Correlation assays were performed using ELISA kits for TNF-α, IL-1β, IL-6, and MCP-1 (Solarbio, Beijing, China). Finally, the OD value at 450 nm was measured with a microplate reader (Bio-Rad, Hercules, CA), and the corresponding concentration was calculated.

### Luciferase reporter assay

Software circBase and StarBase v2.0 were used to predict the putative binding sequence of circ_ANRIL and miR-622. A linear sequence containing the predicted miR-622 binding site of circ_ANRIL (CAGACUGG) was amplified from human genomic DNA and cloned into psiCHECK-2 vector (Promega, Madison, WI, USA) to construct a wild-type luciferase reporter construct (circ_ANRIL_Wt). Site-directed mutagenesis was simultaneously performed using the QuikChange II site-directed Mutagenesis kit (Agilent Technologies, Inc., Santa Clara, CA, USA) to synthesize a mutant construct (circ_ANRIL_Mut) with the corresponding mutant seed sequence. Subsequently, HBMECs were co-transfected with 200 ng of the luciferase reporter plasmid and 50 nM of the miR-622 mimic or miR-NC using Lipofectamine 2000 (Life Technologies). At 48 h after transfection, the firefly and renila luciferase activities were examined by using a Dual Luciferase Reporter Assay System (Promega, Madison, WI, USA). Relative luciferase activity was determined with renilla luciferase as normalization.

### Statistical analysis

Statistical analyses were performed using the Statistical Product and Service Solutions (SPSS, Inc., Chicago, Illinois, USA). Data are presented as mean ± standard deviation (SD). Statistical significance was determined by one-way analysis of variance (ANOVA) or a two-tailed Student’s *t*-test. *p *< 0.05 was considered statistically significant.

## Results

### circ_ANRIL was upregulated in OGD/R-treated HBMECs

**Fig. 1:**
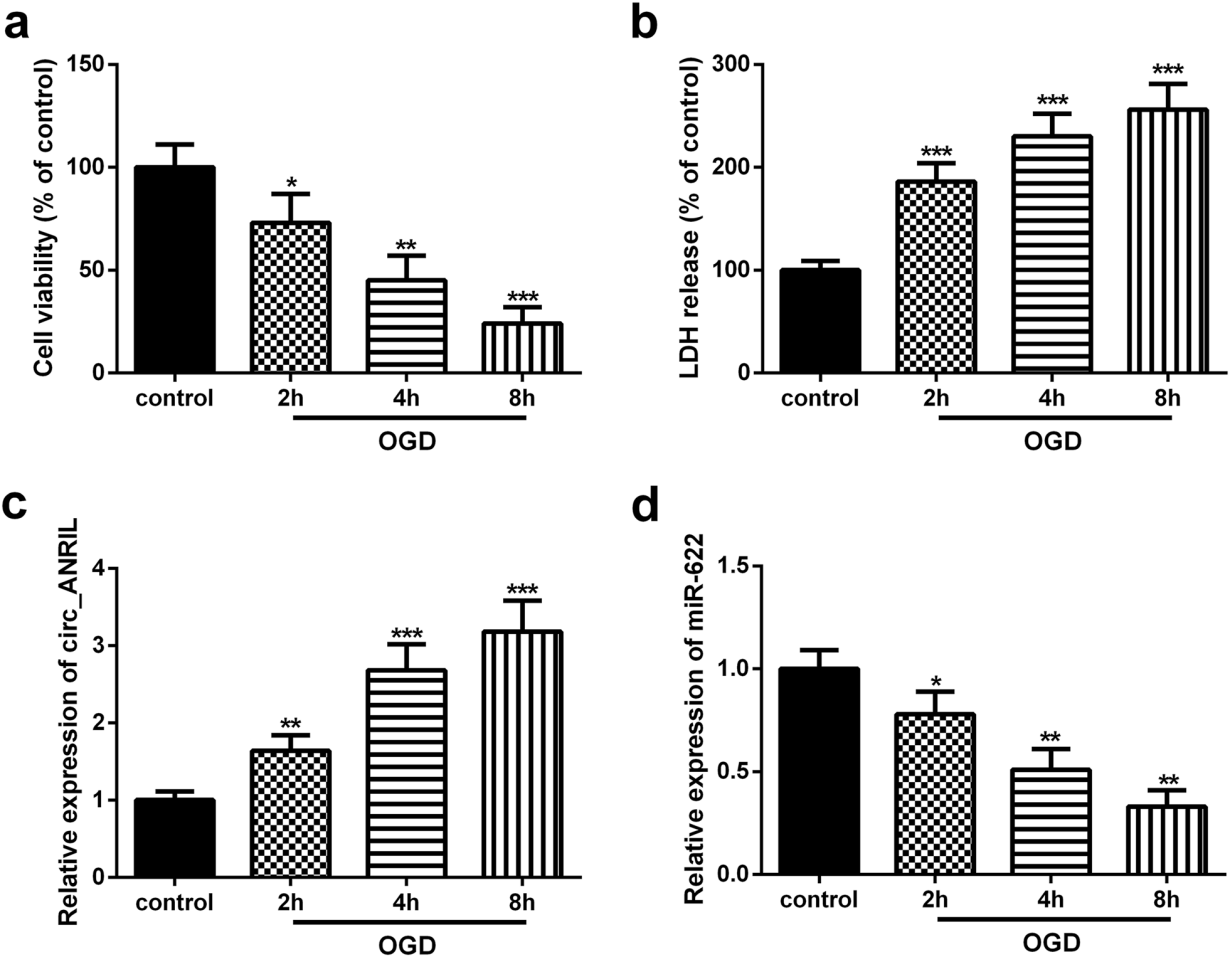
Circ_ANRIL is up-regulated in OGD/R-treated HBMECs. **a** The effect of OGD/R treatment on cell proliferation viability was measured by CCK-8 assay. **b** The OGD/R treatment induced cytotoxicity was analyzed by LDH assay. **c**, **d** The expression levels of circ_ANRIL and miR-622 were detected by qRT-PCR. *p < 0.05, **p < 0.01, ***p < 0.001. *n *= 3

Exposure to OGD/R can effectively simulate the physiological and pathological processes of CI/R [21]. Here, HBMECs were exposed to OGD for 2, 4, and 8 h, and then reoxygenated for 24 h to simulate CI/R in vitro. The Cell Counting Kit (CCK-8) assay showed that OGD/R treatment significantly inhibited the viability of HBMECs (Fig. 1a). Additionally, Lactate dehydrogenase (LDH) release in OGD/R-treated HBMECs was significantly higher than that in control cells (Fig. 1b). These results confirmed that exposure to OGD/R induced cellular damage in vitro.

Next, quantitative real time polymerase chain reaction (qRT-PCR) was performed to detect the expression of circ_ANRIL in OGD/R-treated HBMECs. Results showed that circ_ANRIL expression in OGD/R-induced HBMECs was significantly up-regulated (Fig. 1c), and its expression gradually increased with the prolongation of OGD treatment time. Furthermore, the expression of miR-622 in OGD/R-induced HBMECs was significantly down-regulated compared to control group (Fig. 1d).

### circ_ANRIL aggravates OGD/R-induced cell damage

**Fig. 2:**
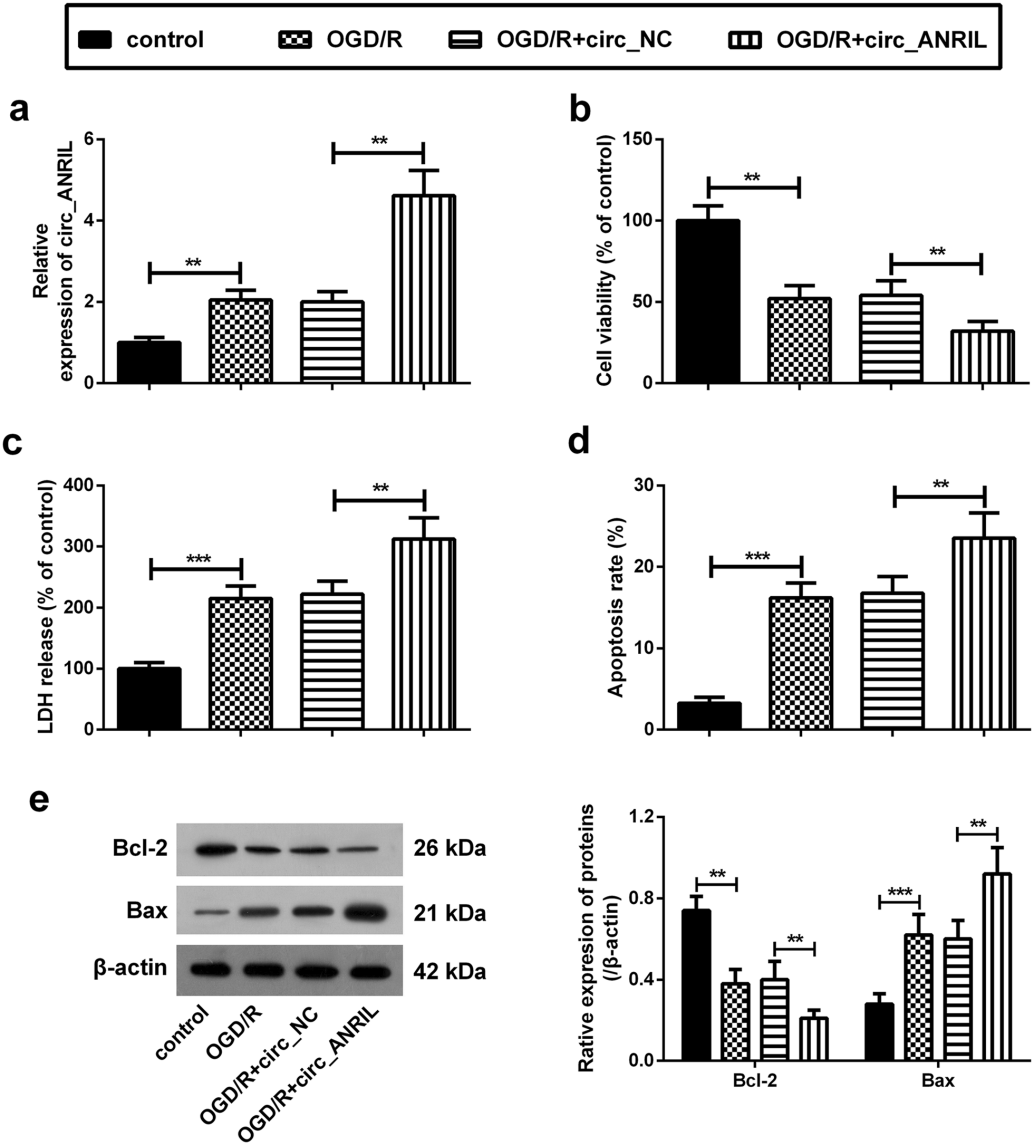
circ_ANRIL aggravates OGD/R-induced cell damage. **a** Upregulation of circ_ANRIL was induced by transfection with circ_ANRIL overexpressing plasmid. The expression of circ_ANRIL in OGD/R-treated HBMECs after plasmid transfection was detected by qRT-PCR. **b** The results of CCK-8 assay showed that overexpression of circ_ANRIL significantly aggravated the reduction of proliferation viability induced by OGD/R treatment in HBMECs. **c** The results of LDH assay showed that circ_ANRIL overexpression aggravated OGD/R-induced cell cytotoxicity. **d** The apoptosis rate of OGD/R-induced HBMECs was analyzed by flow cytometry. **e** The expression of Bcl-2 and Bax was analyzed by western blotting. The promotion effect of circ_ANRIL on OGD/R-induced apoptosis was verified by the changes in protein expression of Bcl-2 and Bax. **p < 0.01, ***p < 0.001. *n *= 3

To further explore the role of circ_ANRIL in CI/R injury, circ_ANRIL overexpression and empty vectors were transfected into HBMECs. After transfection, HBMECs were subjected to OGD for 4 h, followed by reoxygenation for 24 h. The results showed that the relative expression of circ_ANRIL in cells transfected with the circ_ANRIL overexpression vector was significantly higher than that in control cells, while circ_ANRIL expression remained unaffected in cells transfected with the empty vector (Fig. 2a).

The results of CCK-8 assay showed that overexpression of circ_ANRIL significantly inhibited proliferation of OGD/R-induced HBMECs (Fig. 2b). Furthermore, LDH release assay showed that circ_ANRIL overexpression aggravated OGD/R-induced cell toxicity (Fig. 2c). Flow cytometry analysis showed that the apoptosis rate of OGD/R-treated cells was significantly higher than that of control cells (Fig. 2d). Further, western blot analysis showed that, compared with that in control cells, B-cell lymphoma-2 (Bcl-2) expression was significantly decreased in OGD/R-treated cells, while Bcl-3 associated X (Bax) expression was significantly increased (Fig. 2e). Simultaneously, overexpression of circ_ANRIL further promoted OGD/R-induced apoptosis (Fig. 2e and f).

### circ_ANRIL aggravates OGD/R-induced inflammatory response

**Fig. 3:**
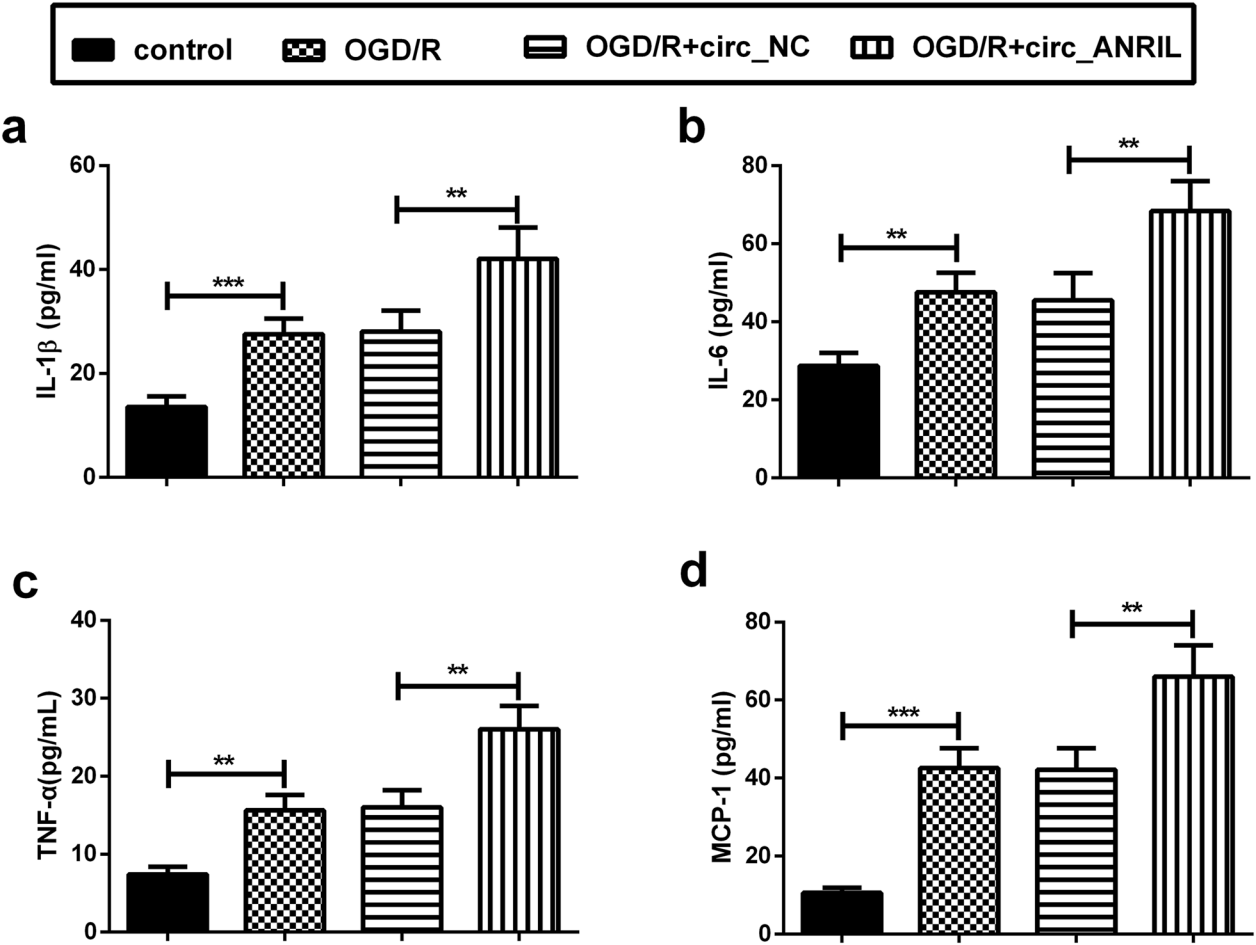
circ_ANRIL aggravates OGD/R-induced inflammatory response. **a–d** The levels of IL-1β, IL-6, TNF-α, and MCP-1 were detected by ELISA. The results showed that circ_ANRIL upregulation promoted inflammatory factor release in OGD/R-treated HBMECs. **p < 0.01, ***p < 0.001. *n *= 3

The up-regulation of inflammatory factors and chemokines constitutes the basis for the transition from ischemic injury to secondary brain injury [22]. Here, we detected a significant increase in the levels of the inflammatory factors, interleukin (IL)-1β, IL-6, tumor necrosis factor (TNF)-α, and monocyte chemotactic protein (MCP)-1, in the supernatant of OGD/R-induced HBMECs. Simultaneously, circ_ANRIL overexpression further increased the secretion of IL-1β, IL-6, TNF-α, and MCP-1 in OGD/R-induced HBMECs (Fig. 3a–d).

### circ_ANRIL targets and regulates miR-622

**Fig. 4:**
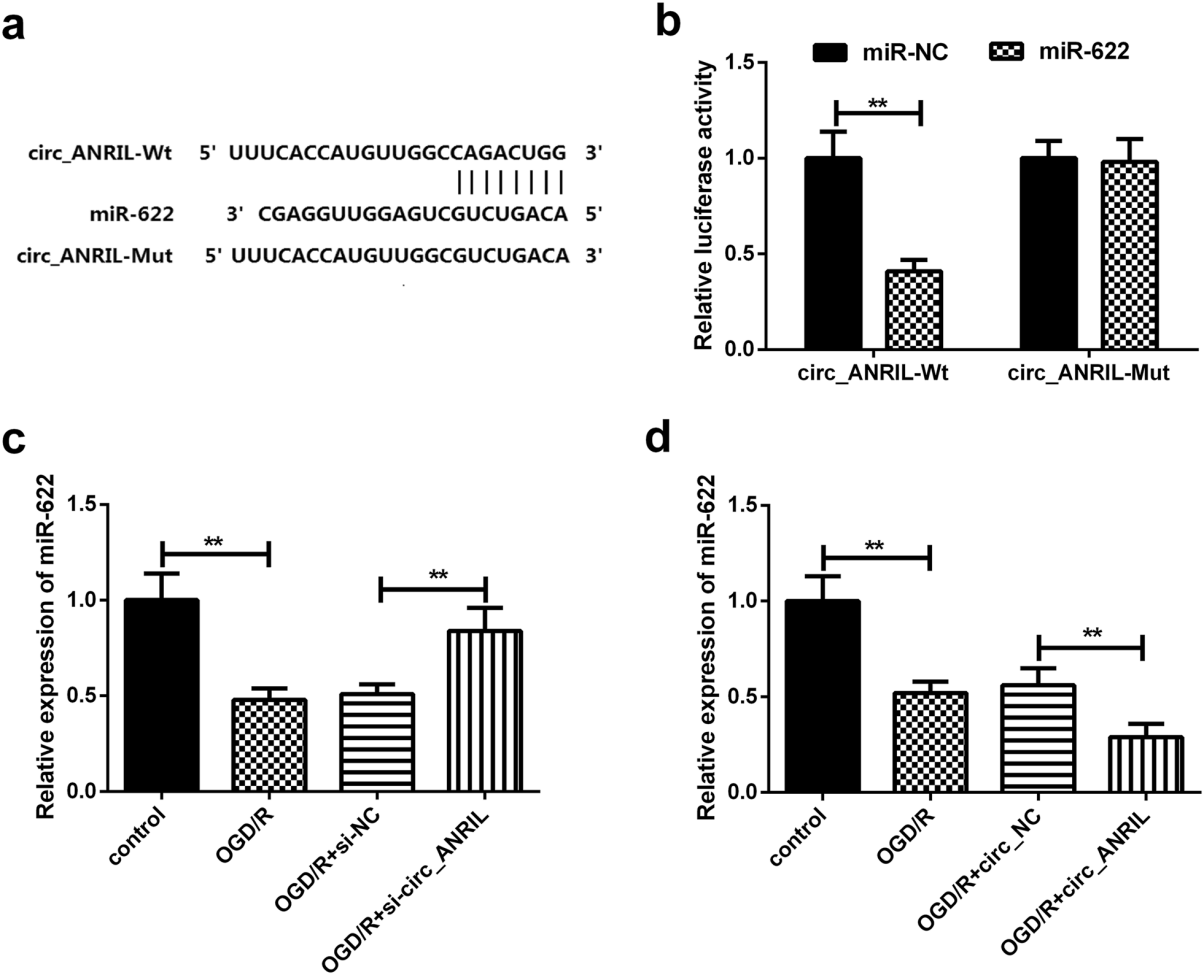
circ_ANRIL targets and regulates miR-622. **a** Paired miR-622 seed sequence and seed recognition site in the 3ʹ-UTR of wild-type (circ_ANRIL-Wt) and (circ_ANRIL-Mut) are indicated. **b** Dual-luciferase reporter assay showed the putative complementary sites between miR-622 and circ_ANRIL. **c**, **d** circ_ANRIL silencing increased miR-622 expression level in OGD/R treated HBMECs. circ_ANRIL upregulation has the opposite effect. **p < 0.01. *n *= 3

Bioinformatics analysis predicted that circ_ANRIL has putative miR-622 binding sites (Fig. 4a). As shown in luciferase assay, miR-622 mimic significantly reduced the luciferase activity of wild-type circ_ANRIL luciferase reporter vector (circ_ANRIL-Wt) in HBMECs compared to miR-NC, whereas the inhibitory effect of 13 miR-622 mimic on luciferase activity was attenuated after co-transfection with circ_ANRIL mutant luciferase reporter vector (circ_ANRIL-Mut) and miR-622 mimic (Fig. 4b). Further analysis revealed that overexpression of circ_ANRIL significantly down-regulated the expression of miR-622 in OGD/R-induced HBMECs, whereas knockdown of circ_ANRIL significantly increased miR-622 expression (Fig. 4c and 4d). These results indicated that circ_ANRIL could act as an miR-622 sponge to negatively regulate the expression of miR-622 in OGD/R-induced HBMECs.

### Silencing of circ_ANRIL attenuates OGD/R-induced cell damage and inflammatory responses by modulating miR-622

**Fig. 5:**
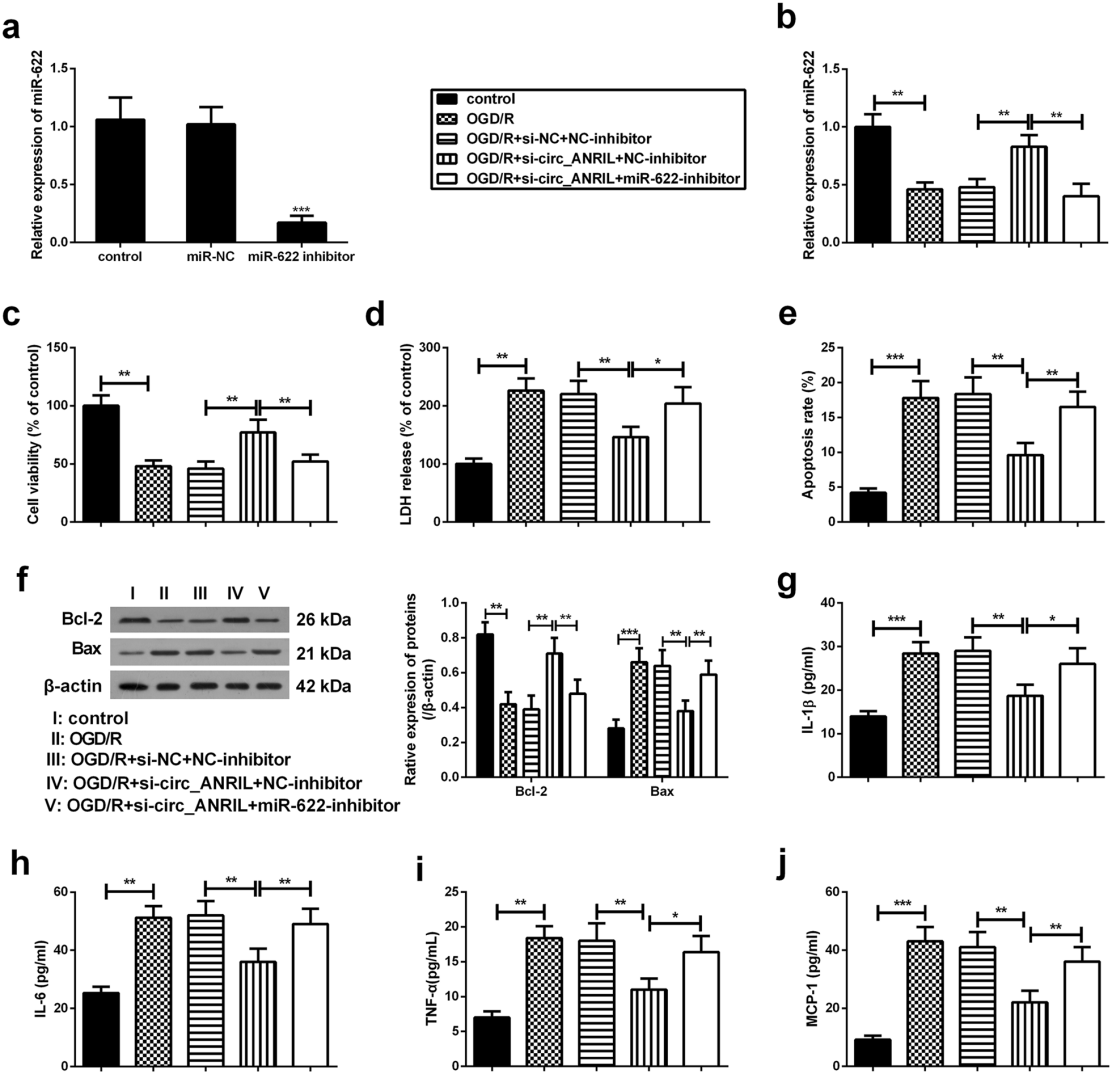
Silencing of circ_ANRIL attenuates OGD/R-induced cell damage and inflammatory responses by modulating miR-622. **a** Downregulation of miR-622 was induced by transfection of miR-622 inhibitor and then its expression was detected by qRT-PCR. si-circ_ANRIL (or si-NC) and miR-622 inhibitor (or NC-inhibitor) were transfected into HBMECs, either alone or in combination. The transfected HBMECs were then exposed to OGD/R. Conventionally cultured HBMECs were used as controls. **b** The expression of miR-622 was detected by qRT-PCR. **c–f** Transfection with si-circ_ANRIL improved cell viability, reduced cytotoxicity and apoptosis, accompanied by the changes in protein expression of Bcl-2 and Bax. **g–j** Silencing of circ_ANRIL inhibits inflammatory factor release in OGD/R-treated HBMECs. Whereas, co-transfection with miR-622 inhibitor counteracted these effects of si-circ_ANRIL on OGD/R-induced HBMECs. *p < 0.05, **p < 0.01, ***p < 0.001. *n *= 3

As shown in Fig. 5a, the expression of miR-622 was markedly decreased in HBMECs transfected with miR-622 inhibitor than that in cells transfected with miR-NC. To elucidate the underlying mechanism of circ_ANRIL in CI/R injury, HBMECs were transfected with circ_ANRIL siRNA (si-circ_ANRIL) or miR-622 inhibitors HBMECs, either alone or in combination. Results showed that transfection with miR-622 inhibitor partly reversed si-circ_ANRIL-induced up-regulation of miR-622 expression (Fig. 5b). In addition, transfection with si-circ_ANRIL alone extenuated OGD/R-induced cell damage, including improved cell viability (Fig. 5c), reduced cell toxicity (Fig. 5d) and apoptosis (Fig. 5e and f), and reduced cellular inflammatory response (Fig. 5g–j). More importantly, transfection with miR-622 inhibitor attenuated these effects of si-circ_ANRIL on OGD/R-induced cell toxicity, apoptosis, and inflammatory responses. These results further confirmed that circ_ANRIL promoted CI/R injury by modulating miR-622 expression.

### Silencing of circ_ANRIL inhibits the activation of the nuclear factor (NF)-κB pathway by regulating miR-622

**Fig. 6:**
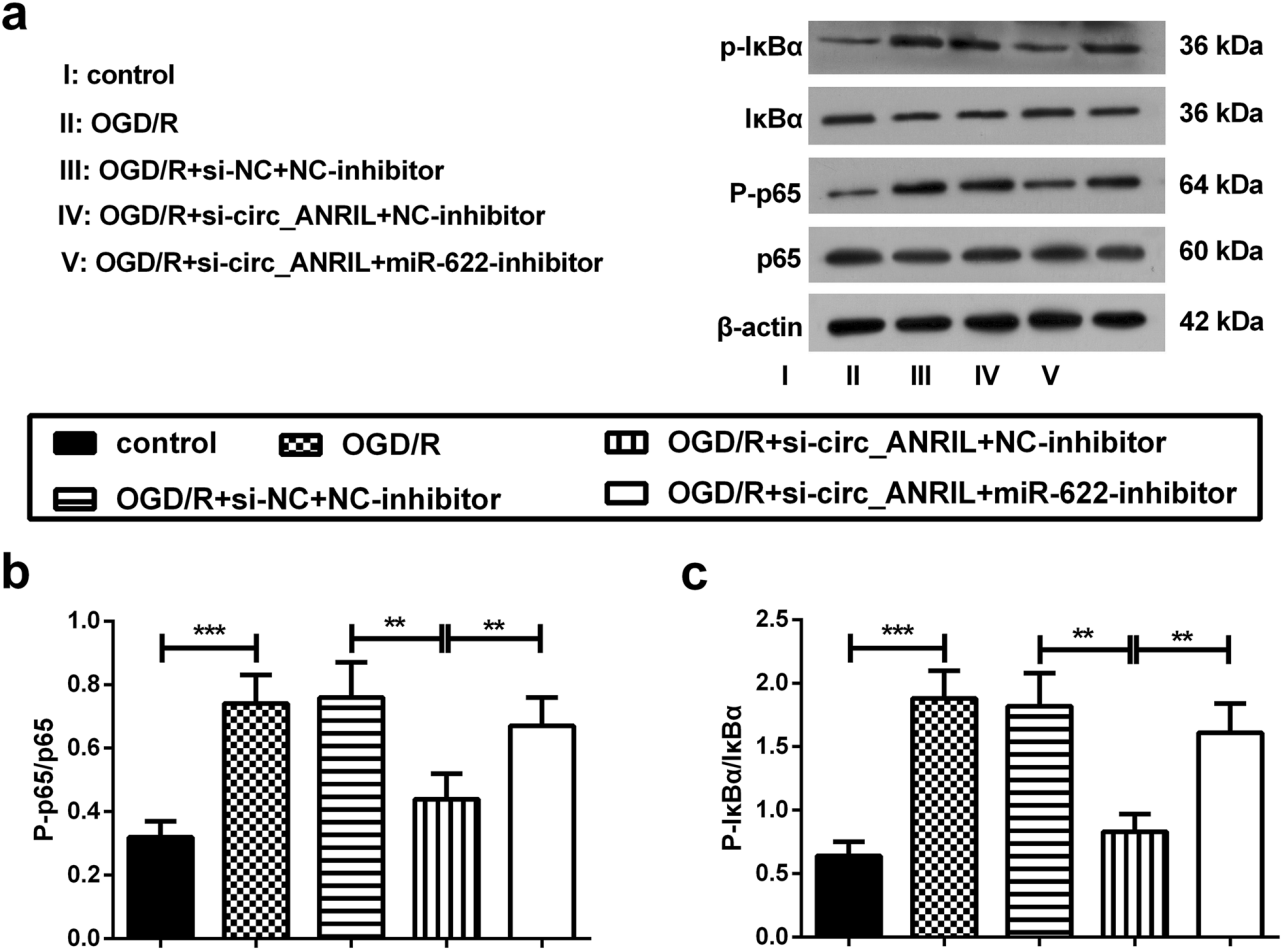
Silencing of circ_ANRIL inhibits the activation of the NF-κB pathway by regulating miR-622. **a** The phosphorylation levels of IκBα and p65 was measured by western blotting. Representative images were presented. **b**, **c** The relative protein expression levels were normalized using β-actin. Results are expressed as mean ± SD. *p < 0.05, **p < 0.01, ***p < 0.001. *n *= 3

Next, we analyzed the expression of the NF-κB pathway-associated proteins by western blotting to further elucidate whether circ_ANRIL affects OGD/R-induced apoptosis and inflammatory responses by stimulating downstream signaling pathways. The results showed that the phosphorylation of p65 and IκBα was significantly increased in OGD/R-induced HBMECs, suggesting that OGD/R treatment induced NF-κB pathway activation in HBMECs. Furthermore, silencing of circ_ANRIL inhibited OGD/R-induced phosphorylation of p65 and IκBα; however, this effect was abrogated by inhibition of miR-622 (Fig. 6a–c). These results indicate that silencing of circ_ANRIL blocks the NF-κB pathway by up-regulating the expression of miR-622 in OGD/R-induced HBMECs.

## Discussion

CI/R injury can cause permanent damage to the brain tissue. Recent studies have shown that circRNA plays an important role in the pathological process of ischemic brain damage [7], Alzheimer’s disease [23], Parkinson’s disease [24], and brain tumors [25]. In the present study, we investigated the role of circ_ANRIL in CI/R injury. Our data showed that the expression of circ_ANRIL in OGD/R-treated HBMECs was significantly up-regulated, while that of miR-622 was down-regulated. Silencing of circ_ANRIL attenuated OGD/R-induced cell toxicity, apoptosis, and inflammatory responses. More importantly, the NF-κB pathway was involved in the regulatory effect of circ_ANRIL/miR-622 axis on OGD/R-induced cell injury.

circRNAs are abundantly expressed in the mammalian brain. Recent studies have reported significant changes in the expression of several circRNAs following ischemic stroke [26]. Liu et al. [27] observed a significant change in the expression of 1024 circRNAs in a CI/R mouse model, of which 914 circRNAs were significantly up-regulated and the remaining 113 were significantly down-regulated. Lin et al. [7] showed that the expression of 3 circRNAs was up-regulated in OGD/R-induced H22 cells, while that of 12 circRNAs was down-regulated. Exploring the role of circRNAs in CI/R injury may have important implications for the discovery of new therapeutic targets. circ_ANRIL, a type of circRNA, has been shown to be associated with atherosclerosis [9, 10]. It has been reported that circ_ANRIL is involved in atherosclerosis by regulating apoptosis and inflammation [10, 11]. However, no studies have evaluated the effect of circ_ANRIL on CI/R injury. Here, our data revealed HBMECsthat the expression of circ_ANRIL increased in OGD/R-induced HBMECs with an increase in OGD treatment time. Simultaneously, circ_ANRIL overexpression promoted OGD/R-induced cell toxicity and apoptosis, and aggravated inflammatory response. These findings suggest that silencing of circ_ANRIL may help improve CI/R-induced brain damage.

CircRNAs can act as miRNA sponges to indirectly regulate the expression of miRNA target genes by competitively binding to miRNAs [28]. In a previous study, Liu et al. [27] selected mmu_circ_40001, mmu_circ_013120, and mmu_circ_40806 and constructed an interaction network of circRNAs-miRNAs-target genes, confirming that changes in circRNAs are associated with genes involved in brain injury and repair. Furthermore, through bioinformatics, GO, and KEGG pathway analyses, Lin et al. [7] found that mmu_circ_015947 participates in apoptosis-, metabolism-, and immune-related pathways by targeting certain miRNAs, all of which are related to the pathogenesis of CI/R. Here, our data demonstrated that miR-622 is regulated by circ_ANRIL and is a direct target of circ_ANRIL. We further investigated whether circ_ANRIL regulated CI/R injury by regulating miR-622. Results showed that circ_ANRIL silencing improved OGD/R-induced apoptosis and inflammatory responses, whereas treatment with miR-622 inhibitor attenuated this effect. These findings indicate that circ_ANRIL is involved in CI/R injury by acting as an miR-622 sponge.

Activation of NF-κB is thought to be critical to mediate or aggravate inflammatory injury following CI/R [29]. Targeted genetic destruction of NF-κB activation in HBMECs has been shown to protect against ischemic stroke by inhibiting the synthesis of inflammatory and adhesion molecules in endothelial cells [30, 31]. Moreover, inhibition of NF-κB activation was shown to reduce cerebral ischemic injury in CI/R rats, which can be attributed, at least in part, to the reduction of inflammation and apoptosis following ischemic injury [32]. It has been reported that silencing of circ_ANRIL protects HK-2 cells from lipopolysaccharide (LPS)-induced inflammatory damage by inhibiting the activation of the NF-κB and c-Jun N-terminal kinase (JNK)/p38 pathways [33]. Here, our results also revealed that silencing of circ_ANRIL inhibited the activation of the NF-κB pathway in OGD/R-induced HBMECs by sponging miR-622. These findings suggest that the NF-κB pathway may be involved in mediating the function of circ_ANRIL in OGD/R-induced apoptosis and inflammatory responses.

## Conclusions

In conclusion, circ_ANRIL knockdown improved OGD/R-induced cell apoptosis and inflammatory response in OGD/R-induced HBMECs by sponging miR-622, a process involving NF-κB pathway. These data displayed the crucial role of circ_ANRIL in OGD/R-induced cell damage in vitro. However, the precise role of circ_ANRIL in CI/R injury requires further investigation using in vivo animal models.

## Data Availability

All data generated or analyzed during this study are included in the published article.

## Abbreviations

CI/R: Cerebral ischemia–reperfusion
circRNA: Circular RNA
ANRIL: Antisense non-coding RNA in the *INK4* locus
OGD/R: Oxygen–glucose deprivation and reoxygenation
HBMECs: Human brain microvascular endothelial cells
miR-622: MicroRNA-622
IL-1β: Interleukin-1β
TNF-α: Tumor necrosis factor-α
MCP-1: Monocyte chemoattractant protein-1
NF-κB: Nuclear factor-κB
